# The Effect of *Sargassum siliquastrum* Supplementation on Growth Performance, Cecal Fermentation, Intestine Histomorphology, and Immune Response of Japanese Quails

**DOI:** 10.3390/ani12040432

**Published:** 2022-02-11

**Authors:** Salma H. Abu Hafsa, Ayman A. Hassan

**Affiliations:** 1 Livestock Research Department, Arid Lands Cultivation Research Institute, City of Scientific Research and Technological Applications, New Borg El-Arab, Alexandria 21934, Egypt; 2 Animal Production Research Institute, Agricultural Research Center, Giza 12619, Egypt; aymanan19@hotmail.com

**Keywords:** *Sargassum siliquastrum*, performance, cecal fermentation, histomorphology, antioxidant status, immune response, Japanese quail

## Abstract

**Simple Summary:**

Market demand is predicted to rise as the growing scope of seaweed-based applications, as feed additives for poultry, expands. Seaweeds are a rich source of essential nutrients and bioactive substances, including polysaccharides and trace elements. Seaweeds also have prebiotic properties that help birds perform better and could be utilised instead of antibiotics. Only a few studies have examined the effect of brown seaweed (*Sargassum siliquastrum*) supplementation on bird performance, but to our knowledge, there have been no studies regarding the effect of *S. siliquastrum* supplementation on the bird’s cecal fermentation and microbial populations. Hence, this research aimed to evaluate the impact of *S. siliquastrum* supplementation on performance, cecal fermentation, and microbial populations, as well as on the immunological response in Japanese quails. The results indicated that the studied *S. siliquastrum* supplement improved the performance, cecal fermentation, microbial populations, intestinal morphology, and immunological response in Japanese quails. Hence, this research implies that a dietary *S. siliquastrum* supplement for quails could be a viable feed additive alternative.

**Abstract:**

This study aimed to investigate the dietary effects of *Sargassum siliquastrum* on the growth performance, nutrient digestibility, cecal fermentation, microbial populations, antioxidant status, immune response, and intestine histomorphology of Japanese quails. A total of 450 Japanese quails, aged 7 days, weighing 27.35 ± 0.23 g, were randomly distributed to three dietary groups in a 42-day feeding experiment. Five replicates were prepared per group, with each replicate consisting of 30 chicks in a cage. The three dietary groups consisted of a basal diet (0% supplementation, which was the control) and diets supplemented with 1% and 2% of *S. siliquastrum*. The results showed that the *S. siliquastrum*-supplemented groups and the control group had similar final body weight (FBW), average body gain (ADG), and average feed intake (ADFI). However, the *S. siliquastrum*-supplemented groups had a better feed conversion ratio (FCR), as well as a lower mortality rate, compared to the control group. *S. siliquastrum* supplementation improved the nutrient digestibility of dry matter (DM), organic matter (OM), crude protein (CP), and crude fiber (CF) (*p* < 0.05). The *S. siliquastrum*-supplemented groups exhibited the heaviest empty intestine and cecum weights, as well as the longest intestinal and cecal length. Furthermore, the total volatile fatty acid (VFA) and the propionic acid concentrations increased significantly in quails fed *S. siliquastrum*-supplemented diets (*p* < 0.05), although the concentration of NH_3_-N decreased (*p* < 0.05). The dietary inclusion of *S. siliquastrum* had a beneficial effect on cecal microbial populations, where the *Lactobacillus sp*. counts increased, and the *E. coli* and *Clostridium perfringens* counts decreased. The histopathological examination of the duodenum confirmed that *S. siliquastrum* dietary supplementation enhanced the height and width of the villi. Quails fed *S. siliquastrum*-supplemented diet exhibited the highest total antioxidant capacity, superoxide dismutase, glutathione peroxidase, and glutathione reductase activities, but the thiobarbituric acid reactive substance was decreased (*p* < 0.05). Serum IgA, IgG, and IgM concentrations increased considerably (*p* < 0.05) in *S. siliquastrum*-supplemented groups. In conclusion, *S. siliquastrum* supplementation in the diet of Japanese quail can provide beneficial effects on performance, cecal fermentation, beneficial bacteria populations, and the immune response, and could be considered as an alternative feed additive in poultry production.

## 1. Introduction

There has been a growing trend towards the use of natural feed additives to improve performance and maintain the health of birds. Many researchers are currently focusing their efforts on discovering viable alternatives for achieving better production and profitability. In the poultry industry, “friendly additives” must be sought to replace the frequent use of vaccinations, drugs, and antibiotics [1]. Therefore, the use of probiotics, prebiotics, herbal powders, and algal products in poultry diets, to improve growth and reproductive attributes, as well as innate immunity that provides adequate protection to birds, has become popular as an alternative to antibiotics worldwide in recent years [2,3,4,5]. The genus Sargassum is a tropical and sub-tropical brown seaweed, comprising 150 species [6]. When compared to red and green seaweeds, brown seaweeds exhibited good antioxidant activities, according to Seenivasan [7]. Phenolic compounds, which range from 20 to 30% of the dry weight of brown seaweeds [8], were one of the most potent antioxidants [9]. Brown seaweeds contain sulphated polysaccharides, proteins, omega-3 polyunsaturated fatty acids, pigments, minerals, and vitamins, in addition to their prebiotic properties [10,11,12]. Seaweeds have been used in poultry to improve the immunological status, reduce the microbial burden in their digestive tract, and increase their growth performance [13]. Seaweeds contain a variety of active compounds, including carotenoids, vitamin B_12_, vitamin C, thiamine, riboflavin, and pyridoxine, which can be used to improve the performance of layers and broilers [13,14]. These seaweed components have been evaluated as feed additives to help broilers perform better by preventing pathogenic bacteria from colonising the small intestine, stimulating beneficial bacteria, improving intestinal architecture, enhancing the antioxidant status, and increasing the immunological response [15,16,17]. Previously, Wiseman [18] reported that feeding broiler chickens 3% sun-dried brown seaweed improved their body weight but did not affect their cecum weight. Incorporating 0.5 brown seaweed into a broiler diet resulted in an increased body weight gain, enhanced immunological response, and a lower mortality rate, as compared to the control diet [19]. Adding 5% brown seaweed to the diet of pigs increased their propionic and butyric acid levels, according to Hoebler [20]. Laminarin, a beneficial component found in brown seaweed, improved mucosal absorption and butyrate synthesis, according to Deville et al. [21]. The dietary inclusion of seaweed has been shown to improve bird health and feed efficiency by increasing the abundance of beneficial gut bacteria and by strengthening the host’s innate immune system [22]. A basal diet supplementation with 100 and 200 mg/kg of a fucoxanthin extract (a brown seaweed derivate) increased catalase (CAT) and superoxide dismutase (SOD) activities, as well as glutathione (GSH) levels. Fucoxanthins can be used to regulate antioxidant metabolism and improve the immune system of broilers [16]. Therefore, this study aimed to evaluate growth performance, cecal fermentation, microbial populations, duodenal histomorphology, antioxidant status, and the immunological response in growing Japanese quails fed a *S. siliquastrum*-supplemented diet.

## 2. Materials and Methods

The research protocol was permitted by the Animal Care and Use Committees of the Scientific Research and Technological Applications (Protocol No. 52-3U-12021), Alexandria, Egypt.

### 2.1. Collection and Preparation of Brown Seaweed (Sargassum siliquastrum)

Brown seaweed (*Sargassum siliquastrum*) was hand-picked from the Red Sea near Hurgada with the help of the National Institute of Oceanography and Fisheries—Hurghada Branch, Egypt. Before being sundried for 2 to 3 days, the collected marine *S. siliquastrum* was adequately washed and rinsed three times in freshwater to remove sand, debris, and other extraneous matter attached to the thalli. The dried samples were ground into a fine powder and were stored in airtight bags for further chemical analyses. *S. siliquastrum* was evaluated as a feed supplement in a powder form, and the chemical composition of the *S. siliquastrum* sample is illustrated in Table 1.

### 2.2. Experimental Design, Birds, and Diets

A total of 450 Japanese quails, at seven days of age, with an average body weight of 27 ± 0.23 g, were randomly allocated to one of three dietary treatments for a five-week experiment. The three dietary treatments were a basal diet, supplemented with 0%, 1%, or 2% *Sargassum siliquastrum*. Each treatment consisted of 150 unsexed quails, with five replications, with 30 unsexed quail chicks each. Quails were reared in wire battery cages (W × H × L cm: 50 × 35 × 95) in a well-ventilated room and maintained under the same conditions, with 23 h of light to 1 h of darkness. The quails had free access to water and feed ad libitum throughout the experiment. The experimental diet was formulated according to the NRC [23]. The composition of the experimental diet is shown in Table 2.

### 2.3. Growth Performance

The weights of individual quails were recorded every week to determine the final body weight (FBW). The amount of feed consumption and residual in each cage was recorded daily to evaluate the average daily feed intake (ADFI). The average daily gain (ADG) and the feed conversion ratio (FCR; g feed/g gain) were calculated. The mortality rate was recorded daily and at the end of the experiment the percentage was recorded for each group.

### 2.4. Nutrient Digestibility

On the last week of the experiment, six quails (three males and three females) were selected from each replicate per treatment and were placed in individual battery cages for the digestibility study. The quails were allowed to acclimate for 2 days, then the feed consumption was recorded, and feces were collected every day before feeding in the morning for 5 consecutive days. Once collected, all quails were removed from the excreta, and the cleaned samples were weighed and oven-dried at 70 °C for 48 h, before being ground and stored for chemical analysis. Diet and feces samples were analysed for dry matter (DM), organic matter (OM), crude protein (PC), crude fibre (CF), neutral detergent fiber (NDF), and acid detergent fiber (ADF). AOAC [24] procedures were used to determine the CP (Method No. 954.01) and ash (Method No. 942.05) contents. The ether extract (EE) was determined using petroleum ether as an extracting agent (40–60 °C) according to the Soxhlet extract method (Method No. 930.09) [24]. The NDF and ADF contents were determined according to the method described by van Soest [25]. The calculation for the nitrogen-free extract is: %NFE = 100% − (%EE + %CP + %Ash + %CF).

### 2.5. Some Digestive Tract Characteristics 

At the end of the feeding trial, 30 quails (15 males and 15 females) from each treatment were slaughtered, to determine the weights and lengths of the intestines and cecum of the Japanese quails. The gut intestinal tract (GIT), from the esophagus to the cloaca, was carefully excised. Any digesta remaining in the whole GIT and the cecum were emptied by gentle pressure. The length (cm) of the whole GIT was measured, as well as the cecum. The GIT’s full and empty weights, and the cecum (g/g body weight), were expressed as a percentage of the body weight.

### 2.6. Cecal Microbes and Fermentation Traits 

At the end of the experiment, four quails were euthanized from each replicate and the cecum was removed to determine the cecal microbial counts (two quails/replicate) and cecal fermentation (two quails/replicate). One gram of cecum content was transferred to 9 mL of 0.1% peptone water (Oxoid, Basingstoke, UK), and was homogenised to enumerate the cecal bacteria. Ten-fold dilutions of each sample were performed with buffered peptone water and were directly inoculated on de Man–Rogosa–Sharpe (MRS) agar for the total aerobic bacterial, anaerobic bacterial, and *Lactobacillus* sp. Counts, and were incubated anaerobically at 37 °C using gas generating kits (Oxoid) for 48 h. The *E. coli* were subcultured on a MacConkey agar and were incubated aerobically at 37 °C for 24 h. *Clostridium perfringens* were subcultured on a Perfringens agar base (Oxoid) mixed with 400 mg of D-cycloserine/liter and were incubated anaerobically using gas generating kits (Oxoid) at 37 °C for 48 h. Depending on the growth characteristics of the bacterial species, bacterial colonies were counted on dishes using a range of 30–300 cfu/g. The overall population was expressed as the log of cfu/g.

The cecal contents of the two quails used to determine cecal fermentation were squeezed out into clean beakers. Immediately, cecal contents were strained through two layers of sterile gauze and the resultant strained liquors were used to measure pH values using an electronic digital pH meter (GLP 21 model; CRISON, Barcelona, Spain). Thereafter, the contents were centrifuged at 7000× *g* for 10 min at 20 °C. The supernatant fluid was divided into two parts. One part was treated with a solution of 5% orthophosphoric acid (*v*/*v*) plus 1% mercuric chloride (*w*/*v*) (0.1 mL-mL^−1^ sample) for the determination of the total VFA concentration and the individual VFA proportions, while the other was acidified with 0.2 M hydrochloric acid solution (one mL-mL^−1^ sample) to be used for the determination of ammonia nitrogen (NH_3_-N) concentration. The total VFA concentration was measured via steam distillation, according to Eadie et al. [26]. The percentage concentration of VFA was analysed using high performance liquid chromatography (HPLC; Model Water 600; UV detector, Millipore Crop.) according to the method of Mathew et al. [27]. After the results of the percentage concentrations of the particular VFA had been received, the concentrations (mmol·L^−1^) of acetic, propionic, and butyric acids were calculated. cecal NH_3_-N concentrations were measured using spectrophotometry according to Chaney and Marbach [28].

### 2.7. Blood Antioxidant Activity and Immunoglobulin Concentration

At the end of the experiment, blood samples were collected from the slaughtered quails into clean tubes and were immediately centrifuged at 1000× *g* for 20 min at 20 °C. The serum was separated and kept at −20 °C until determination of total antioxidant capacity (TAC), superoxide dismutase activity (SOD), glutathione peroxidase (GPx), glutathione reductase (GR), and thiobarbituric acid reactive substances (TBARS) were calorimetrically determined using commercial kits from Biodiagnostic Company (Giza, Egypt) and a spectrophotometer (Optizen Pop, Mecasys, Korea). Serum concentrations of immunoglobulins (Ig) A, G, and M were determined using kits (Bethyl Laboratories, Montgomery, TX, USA). The ELISA procedure was performed according to the manufacturer’s protocol, and the absorbance was measured at 450 nm.

### 2.8. Histopathology Measurements

At the end of the experiment, five additional quails from each treatment were euthanized to determine the histopathology analysis. A 2-cm long segment of the duodenum was transected from each quail, and any digesta remaining in these segments was emptied by gentle washing with normal saline before being fixed in a 10% neutral buffered formalin solution. The fixed samples were processed through a normal alcohol dehydration-xylene procedure before being embedded in paraffin. Histological sections were prepared from 5-µm paraffin blocks of samples and were stained using the haematoxylin and eosin (H&E) technique. The measurements of the villi lengths and crypt depths in the samples were performed using an image analysis program Image J software. A number of well-oriented intact crypt–villus units were selected in triplicate for each cross-section in the duodenum. The appearance of the entire lamina propria served as the villus classification criteria. According to Wilson et al. [29], the length of the villus was measured from the tip of the villus to the villus–crypt junction. 

### 2.9. Statistical Analyses

Data were subjected to statistical analyses in a randomized complete block design using general linear model procedures of SAS/STAT (Statistical Analysis System, version 9.3, SAS Institute Inc., Cary, NC, USA) [30]. The obtained data were tested by an analysis of variance with a one-way design to test the treatment at each sampling, according to the following model:

Y_ij_ = μ + T_i_ + Ɛ_ij_

where y_ij_ is the measured value, μ is the overall mean effect, T_i_ is the ith treatment effect, and Ɛ_ij_ is the random error associated with the jth quails assigned to the ith treatment. Significant differences among the treatments were determined at *p* < 0.05. All results are presented as least-squares means.

## 3. Results

### 3.1. Growth Performance

Table 3 shows the effect of two levels of *S. siliquastrum* (1 and 2%) on quail performance. There was no influence on FBW, ADG, and ADFI between the *S. siliquastrum*-supplemented groups and the control group. Supplementing *S. siliquastrum* into the quail diet improved FCR compared to the control group. *S. siliquastrum*-supplemented groups exhibited a lower mortality rate than the control group.

### 3.2. Nutrient Digestibility

The groups treated with *S. siliquastrum* had improved (*p* < 0.05) digestion coefficients for DM, OM, CP, and CF compared to the control group (Table 4). However, the digestion coefficients for NFE did not differ between the treatment groups.

### 3.3. Digestive Tract Characteristics

The relative weight of empty intestine, cecum, and 1 cm each of empty intestine and cecum was significantly higher for quails fed *S. siliquastrum*-supplemented diets (*p* < 0.05), compared to the control group (Table 5). The intestinal and cecum lengths of the *S. siliquastrum*-fed groups were significantly longer (*p* < 0.05) than those of the control group.

### 3.4. Cecal Microbes and Fermentation Traits

The total anaerobic bacteria and *Lactobacillus* counts increased dramatically in the *S. siliquastrum*-treated groups, but the *E. coli* and *Clostridium perfringens* counts decreased significantly (*p* < 0.05) (Table 6). In terms of pH, acetic acid, and butyric acid, there were no significant differences between the treatment groups and the control group. The concentrations of VFA and propionic acid in the *S. siliquastrum*-treated groups were significantly higher (*p* < 0.05), although the concentration of ammonia was dramatically lower (*p* < 0.05). 

### 3.5. Histopathology Measurements

**Figure 1:**
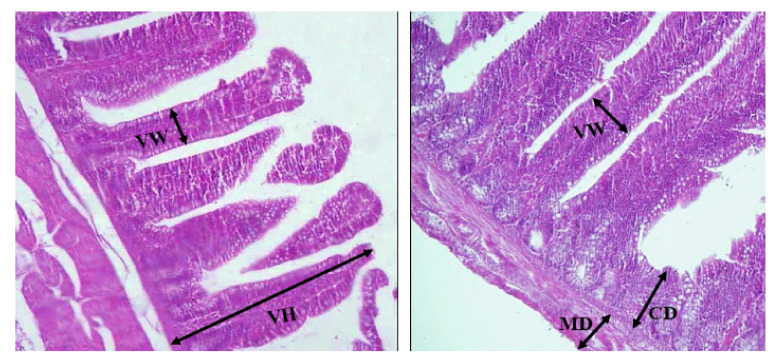
Morphometric measurements of duodenal villus height (VH), villus width (VW), crypt depth (CD) and mucosal depth (MD) of Japanese quail. Scale bar = 100 µm.

Figure 1 and Table 7 illustrate the duodenal histomorphometry in quail chicks. The morphology of the mucosa in the duodenum was altered by the inclusion of *S. siliquastrum* in the diet. Considering the duodenum, quails fed *S. siliquastrum*-supplemented diets had considerably higher average villus heights and widths compared to the control quails. The *S. siliquastrum* supplementation significantly enhanced crypt depth and the villus:crypt ratio (*p* < 0.05), but mucosal depth decreased (*p* < 0.05).

### 3.6. Blood Antioxidant Activity and Immunoglobulin Concentrations

The effect of *S. siliquastrum* supplementation of 1% and 2% on the concentration of antioxidant status and immunoglobulin in Japanese quail serum is shown in Table 8. *S. siliquastrum*-fed quails had the highest TAC, GRx, SOD, and GPx activities, but had the lowest TBARS activities (*p* < 0.05). *S. siliquastrum*-supplemented groups exhibited the highest levels of IgA, IgG, and IgM compared to the control group (*p* < 0.05).

## 4. Discussion

In this study, *S. siliquastrum* supplementation improved the FCR of Japanese quails, which is consistent with the findings of [14]. Choi et al. [19] found that supplementing broilers with seaweed improved their BWG significantly; however, the feed efficiency was not affected. Abu Hafsa et al. [5], on laying quails, and Rizk et al. [31] on laying hens found that the dietary inclusion of marine seaweeds improved their performance. According to Abu Hafsa et al. [32], the growth performance improved significantly in rabbits fed 4% marine seaweeds. Adding seaweed to bird diets improved growth and health, as well as improving intestinal microflora [33]. This can be attributed to the improved immune response, providing adequate protection for the birds. 

Brown seaweeds are rich in a variety of polysaccharides, such as fucans and alginic acids that function as prebiotics, stimulating growth and improving health, according to Wijesinghe et al. [34]. Our results also showed that Japanese quails fed *S. siliquastrum*-supplemented diets exhibited improved nutrient digestibility. Wong and Cheung [35] reported that the presence of phenolic compounds in seaweed, especially phlorotanin in brown seaweed, might affect protein digestion. Similarly, Balasubramanian et al. [36] showed that broilers fed red seaweed-supplemented diets exhibited improved nutrient digestibility. The total digestible nutrients increased significantly in rabbits fed 4% marine seaweed, according to Abu Hafsa et al. [32]. The current study revealed that growing Japanese quails fed *S. siliquastrum* performed as good as, or better than, quails fed a control diet. Seaweed, which is a natural source of many compounds, such as proteins, minerals, fatty acids, polysaccharides, and essential vitamins, can stimulate body metabolism and improve nutrient digestion and absorption, which could explain why the quails in the *S. siliquastrum* groups gained more weight than those in the control group. *S. siliquastrum* improved dietary palatability while simultaneously enhancing digestibility, FCR and intestinal absorption, resulting in increased quail performance and cost efficiency. The beneficial effects in quails can also be ascribed to the rich content of crude proteins and minerals in *S. siliquastrum*. Kumar [14] found that the dietary supplementation of dried *S. wightii* powder at 1%, 2%, 3%, and 4% increased broiler BW, FI, and FCR. Adding 0.25% and 0.5% of sun-dried *A. nodosum* improved broilers’ growth performance [18].

The weight of the full and empty intestines increased significantly when quails were fed *S. siliquastrum*-supplemented diets. In addition, the length of the intestine increased by 9.07% and 9.45%, respectively. The villi’s function is to provide a vastly enlarged surface area for more efficient nutrition absorption. The surface area that is accessible for nutrients to move through affects the absorption efficiency; the more villi there are, the better the absorption is. Despite this, feed intake was unanimously equal, on the whole, in the experiments. Thus, it was easy to demonstrate that the improved BW in *S. siliquastrum*-supplemented birds was attributable to the improved nutrient digestion and absorption, as a result of an increased intestine weight and length. It was observed that the villus height in the small intestine of *S. siliquastrum*-supplemented quails was significantly taller, which is correlated with more efficient nutrient absorption and improved growth performance. Overall, we believe that dietary supplementation with *S. siliquastrum* can improve the growth performance of Japanese quails, as well as their nutrient digestibility, by strengthening their intestinal integrity and immune system. In quails fed the *S. siliquastrum*-supplemented diet, their cecum length increased by 8.13% and 7.70%, respectively, above the control value. These findings could be attributable to the fact that seaweeds have been shown to increase immune function and lower the microbial load in the gastrointestinal system [37].

The supplementation of *S. siliquastrum* into the quail diet increased the abundance of *Lactobacillus* in the cecum, while decreasing *E. coli* and *C. perfringens* concentrations. Macroalgae polysaccharides could be prebiotic ingredients for animal health applications, promoting the growth and/or activity of beneficial gut microbiota, such as *Lactobacillus sp*., which, in turn, confers health benefits to the host by reducing pathogen invasions and disease [38,39]. *Lactobacillus* bacteria inhibit the growth of harmful microorganisms in the gut by producing lactic and acetic acids, which lower the pH of the gut and make it unfavourable for pathogen growth. *Lactobacillus* bacteria also enhance immunity by up-regulating intestinal mucins synthesis, which disrupts pathogen adhesion to the intestinal epithelium, consequently preventing pathogen translocation. These results are consistent with those of [40,41]. The increased count of *Lactobacillus* bacteria that contribute to the development of resistance to *C. perfringens* and *E. coli* colonization through a competitive exclusion mechanism may be responsible for the observed improvements in the microbial community. Seaweed contains a variety of active compounds, including polysaccharides, such as laminarin and fucoidan [42]; proteins such as lectins [43]; phlorotannins [44]; and pigments, such as carotenoids [45] which act as prebiotics in promoting the growth of beneficial bacteria while inhibiting harmful microorganisms, hence, improving overall health [46]. 

The weight of the cecum is an indicator of the fermentation in the cecum. An increase in cecum weight is associated with a rise in the count of beneficial bacteria. [47]. The total VFA, which is a primary end product of microbial fermentation and improves gastrointestinal fluid absorption, is affected by changes in the bacterial community structure. [48]. The concentration of VFA in the intestine is determined primarily by the fermentative substrate and the microbial diversity in the intestine [49]. Supplementing Japanese quails with *S. siliquastrum* increased VFA and propionic acid significantly; however, both acetic and butyric acids were not affected. According to Gomez-Ordonez et al. [50], the propionate concentration in the cecum was increased dramatically when seaweed was included in feed. The greater propionate acid concentrations found in this investigation was consistent with previous findings in broilers [51]. Deville et al. [21] reported that laminarin, a brown seaweed polysaccharide, improved mucosal absorption and butyrate synthesis. Kulshreshtha et al. [47] found that the concentration of VFA, including acetic, propionic, and butyric acids, was substantially greater in red seaweed treatments than in a control. The VFA is an important source of energy for enterocytes, and is also key in signaling molecules for gut health maintenance. In addition, the VFA can indirectly stimulate cell proliferation, resulting in an increase in the weight of the intestine and cecum. [52]. It is, thus, probable that the elevated VFA concentration in this study contributed to the larger intestine and cecum weights. As a result, it’s likely that the higher VFA content in our study led to the increased intestine and cecum weight. 

The antioxidant status of birds is extensively documented as being significant for their resistance to infections, health maintenance, and their productivity [53]. Increases in TAS, SOD, GRx, and GPx, but a decrease in TBARS, were indicative of lower lipid peroxidation levels, according to Abdel-Daim et al. [54]. The *S. siliquastrum*-treated group had significantly lower TBARS levels than in the control group. This supports the findings of Droge [55] who reported that *Spirulina platensis* had a high antioxidant capacity in broilers, which was linked to the presence of β-carotene zeaxanthin, phycocyanin, and allophycocyanin [56]. Superoxide dismutase (SOD), the most significant antioxidant enzyme, is essential for the removal of superoxide anions in animals [57]. The considerable increase in SOD values in the groups supplemented with *S. siliquastrum* could imply a strong association between a microalgae addition and the improved antioxidant capacity, as documented by Droge [55]. It is known that the immunological status of the host has a significant impact on various infection resistance. In addition to protecting the apical surface of the brush border, IgA plays a vital function in gastrointestinal defence and secretion in the gut lumen [58]. Therefore, IgA has the potential to alter the microbial population by targeting those species that the immune system considers harmful [58]. IgG is the main serum antibody in the mucosal immune response [59]. According to our findings, the elevated concentrations of IgA, IgG, and IgM antibodies in the supplemented groups imply that *S. siliquastrum* in the diet can strengthen the immune system for antibody production in Japanese quails. Alginates, the active components of brown seaweed, consist of two types of uronic acids: mannuronic and guluronic [60], which function as prebiotics, as well as immunomodulators that enhance innate immunological resistance in birds and animals [61]. 

## 5. Conclusions

The results of this study demonstrated that *S. siliquastrum* supplementation in the diets of quails enhanced their intestinal health through positively modulating of cecal microbiota and fermentation, as well as improving the feed conversion ratio, nutrient digestibility, stimulated antioxidant status, and the immune response. Therefore, *S. siliquastrum* can be considered an effective feed additive for poultry. 

## Figures and Tables

**Table 1:** The chemical composition of *Sargassum siliquastrum*.

Items	*Sargassum siliquastrum*
Chemical analysis (% on DM basis)	
Organic matter	78.31
Crude protein	17.83
Crude fibre	16.42
Ether extract	2.96
NFE	41.10
Ash	21.68
NDF	41.58
ADF	26.84
ADL	8.11
Hemicellulose	14.73
Cellulose	18.73
Mineral composition, mg/kg:	
Sodium	211.4
Potassium	94.5
Calcium	70.7
Magnesium	188.9
Phosphorus	279.1
Iodine	116.6
Lead	0.05
Cadmium	0.027
Iron	6.37
Copper	0.11
Manganese	0.10
Selenium	0.86
Zinc	0.64

NFE, nitrogen-free extract; NDF, neutral detergent fibre; ADF, acid detergent fibre; ADL, acid detergent lignin.

**Table 2:** Ingredients and chemical composition of the experimental diets.

Ingredients (%)	Starter Phase (1–3 Weeks)	Finisher Phase (4–6 Weeks)
Yellow corn	54.1	60.4
Soybean meal (44% CP)	28.5	22.3
Protein concentrate *	10.00	9.1
Wheat bran	6.00	6.8
Vegetable oil	0.50	0.50
Dicalcium phosphate	0.20	0.20
Vitamin and mineral premix **	0.30	0.30
L-lysine	0.15	0.15
Dl- methionine	0.25	0.25
Total	100	100
Chemical analysis, %		
Crude protein	23.07	20.46
Crude fibre	3.42	3.71
Ether extract	4.73	4.94
Calculated nutritional values		
Metabolizable energy (MJ/kg)	14.73	14.82
Calcium, %	0.83	0.79
Available phosphorus, %	0.33	0.29

* Protein concentrate contains: 52% crude protein, 2.03% crude fibre, 6.17% ether extract, ME 2080 (Kcal/Kg), 1.50% methionine, 2.00% methionine and cystine, 3.0% lysine, 7.00% calcium, 2.93% available phosphorus, and 2.5% NaCl. ** Every 3 kg of mineral and vitamin premix (per ton of feed) contains vitamin A, 12,000,000 IU; vitamin D3, 2,000,000 IU; vitamin E, I0 g; vitamin K3, 1000 mg; vitamin B1, 1000 mg; vitamin B2, 5 g; vitamin B6, 1.5 g; vitamin B12, 10 mg; pantothenic acid, 10 g; niacin, 30 g; folic acid, 1 g; biotin, 50 mg; iron, 30 g; manganese, 60 g; choline chlorite, 10 g; iodine, 300 mg; copper, 4 g; zinc, 50 g; and selenium, 100 mg.

**Table 3:** Effects of *S. siliquastrum* dietary treatment on growth performance in Japanese quails.

Items	Control	*S. siliquastrum*		SEM	*p*-Value
		1%	2%		
Initial body weight, g	27.84	27.04	27.16	0.68	0.782
Final body weight, g	219.54	227.77	231.52	11.94	0.744
Average daily gain, g	4.56	4.78	4.87	0.36	0.836
Average daily feed intake, g	15.52	14.88	14.72	1.26	0.682
Feed conversion ratio	3.40 ^a^	3.11 ^b^	3.02 ^b^	0.08	0.015
Mortality rate	4.00 ^a^	2.00 ^b^	0.66 ^c^	0.02	0.001

^a,b,c^ Means in the same row bearing different superscript letters differ significantly (*p* < 0.05).

**Table 4:** Effect of *S. siliquastrum* dietary treatment on nutrient digestibility in Japanese quails.

Items	Control	*S. siliquastrum*		SEM	*p*-Value
		1%	2%		
Dry matter	76.47 ^b^	78.95 ^a^	79.11 ^a^	0.17	0.011
Organic matter	77.55 ^b^	79.17 ^a^	79.33 ^a^	0.22	0.016
Crude protein	74.44 ^b^	76.11 ^a^	76.35 ^a^	0.21	0.003
Crude fibre	25.61 ^b^	27.88 ^a^	28.79 ^a^	1.21	0.006
Nitrogen-free extract	79.41	80.22	80.41	0.93	0.855

^a,b^ Means in the same row bearing different superscripts differed significantly at (*p* < 0.05).

**Table 5:** Effect of *S. siliquastrum* dietary treatment on weight and length of intestines and cecum in Japanese quails.

Items	Control	*S. siliquastrum*		SEM	*p*-Value
		1%	2%		
Full intestine weight, %	3.89	3.93	3.88	0.31	0.844
Empty intestine weight, %	3.18 ^b^	3.69 ^a^	3.76 ^a^	0.13	0.011
1 cm of empty intestine weight, cm/ g BW	0.049 ^b^	0.052 ^a^	0.053 ^a^	0.009	0.0001
Cecum weight, %	0.79 ^b^	0.92 ^a^	0.93 ^a^	0.01	0.003
1 cm of cecum weight, cm/g BW	0.084 ^b^	0.091 ^a^	0.092 ^a^	0.01	0.0001
Intestinal length, cm	65.16 ^b^	71.07 ^a^	71.19 ^a^	1.07	0.001
Cecal length, cm	9.35 ^b^	10.11 ^a^	10.07 ^a^	0.27	0.002

^a,b^ Means in the same row bearing different superscript letters differ significantly (*p* < 0.05).

**Table 6:** Effect of *S. siliquastrum* dietary treatment on cecal fermentation in Japanese quails.

Items	Control	*S. siliquastrum*		SEM	*p*-Value
		1%	2%		
Bacterial count (log cfu/g)					
Total anaerobic bacteria	5.43 ^b^	5.62 ^a^	5.71 ^a^	0.11	0.015
Total aerobic bacteria	6.41 ^a^	6.11 ^b^	6.05 ^b^	0.08	0.002
*Lactobacillus*	6.77 ^b^	7.06 ^a^	7.11 ^a^	0.04	0.007
*E. coli*	4.84 ^a^	4.22 ^b^	4.06 ^b^	0.18	0.033
*Clostridium perfringens*	2.48 ^a^	2.01 ^b^	1.93 ^b^	0.07	0.019
Cecal fermentation					
pH	6.27	6.22	6.20	0.26	0.822
NH_3_-N (mg/dL)	8.22 ^a^	6.05 ^b^	5.91 ^b^	0.16	0.011
Total VFA (μmol/g)	58.74 ^b^	62.41 ^a^	62.94 ^a^	0.51	0.016
Acetic acid (μmol/g)	64.15	63.95	63.52	0.71	0.739
Propionic acid (μmol/g)	20.73 ^b^	23.82 ^a^	24.26 ^a^	0.49	0.021
Butyric acid (μmol/g)	8.28	8.05	7.89	0.37	0.683

^a,b^ Means in the same row bearing different superscript letters differ significantly (*p* < 0.05).

**Table 7:** Effect of *S. siliquastrum* dietary treatment on intestinal histomorphometry in Japanese quails.

Items	Control	*S. siliquastrum*		SEM	*p*-Value
		1%	2%		
Villus height (μm)	861.84 ^b^	1315.43 ^a^	1317.39 ^a^	45.16	0.008
Villus width (μm)	198.30 ^b^	296.74 ^a^	283.28 ^a^	15.12	0.001
Crypt depth (μm)	119.46 ^b^	263.46 ^a^	266.59 ^a^	7.44	0.001
Villus crypt ratio	7.21 ^a^	4.99 ^b^	4.94 ^b^	1.07	0.003
Mucosal depth (μm)	140.88 ^a^	134.09 ^a^	123.49 ^b^	5.84	0.016

^a,b^ Means in the same row bearing different superscript letters differ significantly (*p* < 0.05).

**Table 8:** Effect of *S. siliquastrum* dietary treatment on blood antioxidant status and immunoglobulin concentration in Japanese quails.

Items	Control	*S. siliquastrum*		SEM	*p*-Value
		1%	2%		
Antioxidant status					
TAC (U/mL)	1.13 ^b^	1.39 ^a^	1.42 ^a^	0.06	0.001
SOD (U/mL)	56.24 ^b^	73.19 ^a^	73.64 ^a^	0.48	0.001
GPx (U/mL)	8.23 ^b^	9.07 ^a^	9.11 ^a^	0.08	0.012
GR (U/mL)	18.51 ^b^	23.66 ^a^	24.16 ^a^	0.61	0.005
TBARS (U/mL)	20.84 ^a^	17.26 ^b^	16.66 ^b^	0.69	0.001
Immunoglobulin concentration					
IgA (mg/dL)	178 ^b^	201 ^a^	218 ^a^	16.64	0.008
IgG (mg/dL)	215 ^b^	255 ^a^	261 ^a^	5.82	0.003
IgM (mg/dL)	142 ^b^	169 ^a^	177 ^a^	7.44	0.001

^a,b^ Means in the same row bearing different superscript letters differ significantly (*p* < 0.05). Total antioxidant capacity (TAC), superoxide dismutase activity (SOD), glutathione peroxidase levels (GPx), glutathione reductase levels (GR), and thiobarbituric acid reactive substances (TBARS).

## Data Availability

The data presented in this study are available on request from the corresponding author.
